# An exploratory study of a multi-species probiotic formulation and markers of health in a real-world oncological cohort in the time of covid

**DOI:** 10.1007/s10787-024-01503-1

**Published:** 2024-06-26

**Authors:** Michael Thomsen, Ravichandra Vemuri, Flavia Huygens, Stephen Clarke, Luis Vitetta

**Affiliations:** 1 Faculty of Medicine and Health, The University of Sydney, Camperdown, Sydney, NSW 2006 Australia; 2 School of Health Sciences, University of Tasmania, Launceston, TAS Australia; 3 School of Biomedical Sciences, Queensland University of Technology, Brisbane, QLD Australia

**Keywords:** Intestinal mucositis, Chemotherapy, Radiotherapy, Intestinal microbiota, Probiotics

## Abstract

**Introduction:**

The efficacy of cancer treatments has links to the intestinal microbiome. Mucositis is a dose-limiting intestinal pro-inflammatory side effect of cancer treatments, that increases the risk of diarrhoea, mucositis, and in severe cases, febrile neutropenia.

**Methods:**

The effect of cancer treatments on Quality of Life (QoL) was assessed using the FACT C questionnaire that included patient wellbeing and gut adverse symptoms (e.g. diarrhoea). Participants rated faecal samples via the Bristol Stool Chart. In addition, bacterial DNA was extracted from faecal samples, sequenced, and taxonomically examined. The incidence / severity of neutropenia was assessed with white blood cell and neutrophil counts. Circulating SCFAs and plasma lipopolysaccharide (LPS) endotoxin levels were recorded and correlated to intestinal mucositis.

**Results:**

Improvement in bowel function, with reduction in constipation and or diarrhoea or absence of significant disturbance to bowel function was recorded in 85% of the participants. One participant developed febrile neutropenia and two developed bowel toxicity during the study, that was unrelated to the test formulation. No significant changes in microbiota alpha- and beta-diversity at the phylum and species levels respectively from baseline to end of study treatment was observed. None of the participants had raised plasma-endotoxin levels from baseline to the first and subsequent treatment cycles for their cancers. Probiotics in this cohort were deemed safe and tolerable. Significant improvement in emotional QoL scores (*p* = 0.015) was reported with increased number of chemotherapy cycles. In a related observational study of exceptional responders to chemotherapy, participants were found to have had a high intake of fruits, vegetables, and fibre possibly indicative of a more balanced intestinal microbiota.

**Conclusion:**

A multi-strain probiotic formulation was safe and tolerated in this chronically ill cohort that were undergoing oncological treatment. The probiotic formulation alleviated diarrhoea, constipation and maintained stool consistency/frequency during the multiple treatments with chemotherapy and radiotherapy. Intestinal dysbiosis that is characterised by decreased microbial diversity and increased pro-inflammatory species was not observed. Probiotic supplementation may have helped reduce dysbiosis during cancer treatments. These improvements may have been critical with the observation that emotional wellbeing was significantly improved from baseline. Hence albeit that the study had limitations, the probiotic intervention provided adjunctive treatment support to the patients. What is of scientifically plausible interest is that probiotics have a long association historically with human hosts and as such ratify their inclusion offering a significant adjunctive therapeutic potential. Future studies warrant larger sample sizes, control groups and should limit recruitment to a largely homogenous group of patients.

## Introduction

The intestines present a collection of bacteria, archae, eukarya and enteric viruses with a high level of complexity and dynamism in its metabolic and immune functions maintaining homeostasis in a symbiotic relationship with the host (Li et al. 2019).

Studies indicate that disturbances in the gut microbiota links bacteria as complicit in a number of metabolic diseases including cancer (Procházková et al. 2023). Over the past 2 decades, it has been reported that 11 bacteria have been labelled as cancer promoting *oncomicrobes* (e.g. *Fusobacterium nucleatum*) (IARC 2012; Humans 2012) with numerous other co-inhabitants in the gut as complicit (e.g. *Bacteroides fragilis*) in cancer progression with ineffective oncological treatment responses (Li et al. 2019; Zhou et al. 2022; Humans 2012). *Oncomicrobes* have been reported to cause an estimated 2.2 million cases per year (~ 13%) of global cancer cases, and epidemiological, molecular mechanisms, and clinical studies have been extensively reviewed (Working-Group-Iarc 2012). Whilst causal evidence of microbial positive influences on cancer biology have emerged, enhanced molecular mechanisms of understanding of such cancer–modulating interactions and impacts on specific cancer treatments provide robust scientific advances of clinical relevance (Yi et al. 2019). Studies continue to report that intestinal bacteria may have important participatory roles in the treatment of tumours. The general development and progression of cancer involves multiple mechanisms. The mechanisms that maintain and then promote proliferation are those that evade cell growth suppression, activate tissue invasion and metastatic pathways, enable replicative immunity, induce angiogenesis, and resist autophagy effectively proliferating and evading the immune system (Hanahan and Weinberg 2011).

Patients receiving cytotoxic and radiation therapy exhibit marked changes in abundance and alpha -diversity in the intestinal microbiota with associated widespread intestinal mucositis manifesting as pain, inflammation, dysphagia, diarrhoea, weight loss, rectal bleeding, and infections (Sougiannis et al. 2021). Mucositis in the gut is a major dose-limiting side effect of chemotherapy and limits nutritional intake and oral function, resulting in patient distress with weight loss and malnutrition (Sougiannis et al. 2021).

The intestinal microbiota is essential to the health of the host, with nutritive functions as well as important effects on non-haematopoietic and haematopoietic structures in the gut maintaining homeostasis (Dethlefsen and Relman 2011). Hence, it is reported that intestinal microbes can impact cancer-treatment responses as well as associated toxicities.

Modulation of the intestinal microbiome has been recently reviewed (Badgeley et al. 2021; Bhat et al. 2022; Zhou et al. 2021). Different modalities have the potential to enable rational microbiome manipulation that could contribute beneficially to cancer treatments. These modalities include data-driven dietary interventions, whole community vaginal and faecal microbiome transfers, postbiotic therapies, pharmaceutical antibiotics eradication of cancer promoting microbes, or with alternative bacteriophage formulations that target commensal bacteria or intramural bacteria and with precision probiotics consisting of gut-directed bacteria at improving colonizable niche (Sougiannis et al. 2021).

In this pilot study we have investigated modulating the intestinal microbiota in patients diagnosed with cancer undergoing chemotherapy and or radiotherapy treatments for patients diagnosed with a cancer, by co-administering to the pharmacotherapy treatment a multi-strain probiotic formulation and recording the highest quintiles of food fibre consumed during the cancer treatments.

**Fig. 1:**
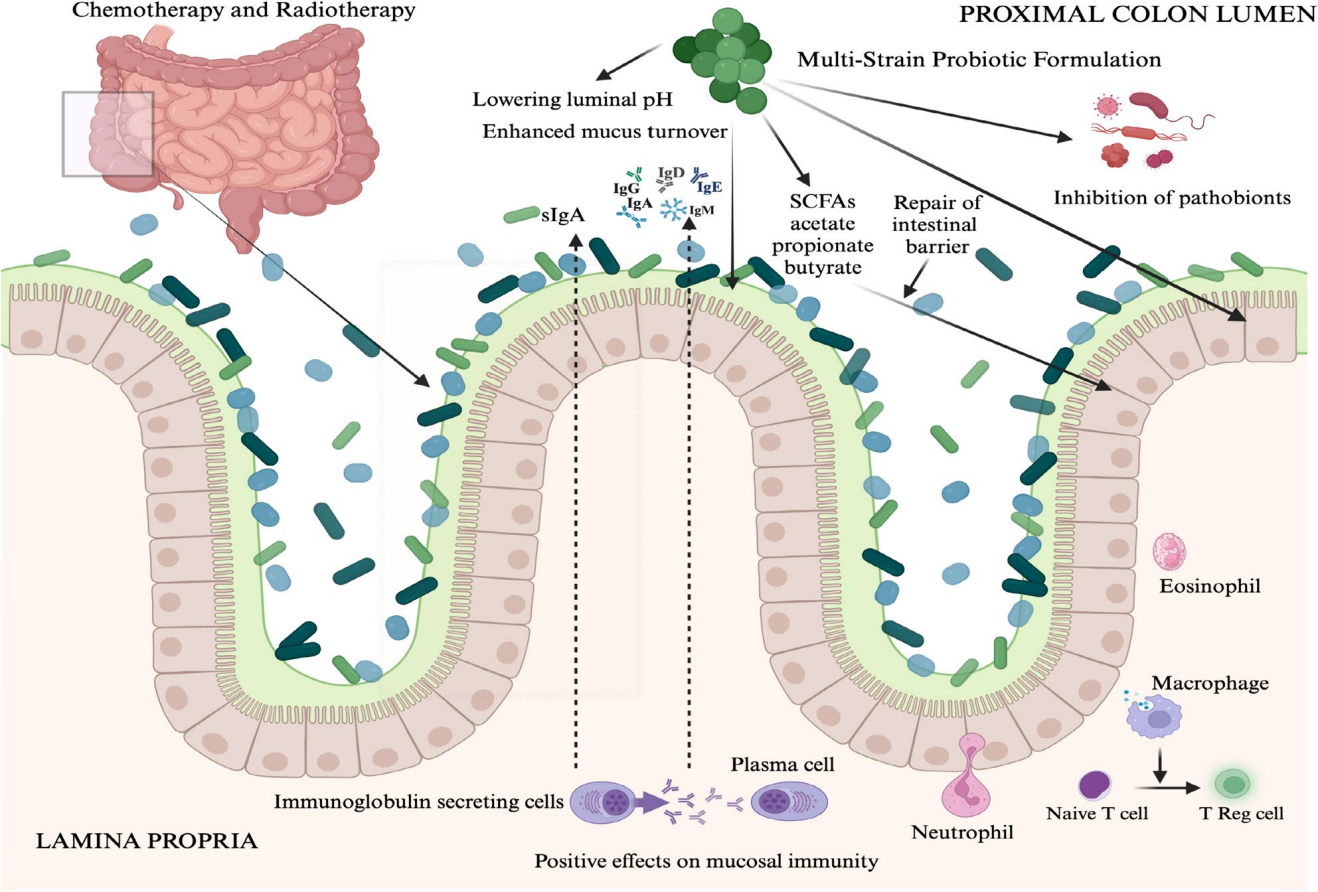
A diagrammatic representation of postulated effects of probiotics in the colon

Probiotic bacteria have been reported to have multiple effects in the intestines (Fig. 1) (Danis et al. 2022). As such in this exploratory study we have considered their adjunctive medicine effects to chemotherapy and or radiotherapy treatments. Thus, supporting the notion that probiotic bacteria will efficaciously ameliorate enteropathies such as diarrhoea and mucositis resultant from chemotherapy and or radiotherapy regimens (Thomsen et al. 2018). Furthermore, we posit that an important benefit that warrants a further focussed research effort is the administration of adjuvant probiotics specifically from species from the *Lactobacillus* and *Bifidobacterium* genera that have been demonstrated to mitigate intestinal inflammatory responses (Cazorla et al. 2018; Thomsen et al. 2018) resulting in a reduced incidence of febrile neutropenia.

## Methods

### Pilot trial design

This was a single-group exploratory safety and tolerability study conducted with patients attending the Northern Cancer Institute in Sydney and the Icon Cancer Centre in Hobart, Australia. Changes in intestinal dysbiosis assessed by faecal testing and changes in systemic inflammatory markers were assessed by blood tests. The study sought to enrol 30 men and women into the pilot trial on satisfying inclusion and exclusion criteria and upon signing/written informed consent.

At baseline, participants were required to provide:

1. Faecal samples for FMA (faecal microbial analysis) to assess the microbiome characterisation.
2. Non-fasting blood samples, to be collected for gut permeability biomarkers including serum lipopolysaccharide levels (LPS) to evaluate intestinal dysbiosis.

### Ethical approval

The National Institute of Integrative Medicine Human Research Ethics Committee (EC00436) granted protocol approval coded as NIIM HREC Reference number: 0043E_2017 on 26th of November 2018. This study was carried out according to the Declaration of Helsinki. The study was conducted in compliance with all stipulations of the protocol, the conditions of ethics committee approval, the NHMRC National Statement on Ethical Conduct in Human Research (2007) and the Note for Guidance on Good Clinical Practice (CPMP/ICH-135/95), and the International Conference on Harmonisation Good Clinical Practise guidelines. The trial was registered on the Australian New Zealand Clinical Trial Registry (ACTRN12618000927224).

### Intervention

A daily dose of four capsules of a multi-strain probiotic formulated and supplied by Medlab Clinical Ltd (MultiBiotic™) was administered by clinical trial participants following a baseline faecal sample collection. Faecal and blood samples were also collected from participants at a midpoint of their cancer treatment and at the end of treatment.

The investigational product was labelled as MultiBiotic™ (i.e. with a Therapeutic Goods Administraiton listing of ARTG 227562) described as a multi-genera and multi-species probiotic formulation developed by research scientists at Medlab Clinical Ltd positing to provide support for gastrointestinal health maintenance. Multibiotic™ contains three species of *Lactobacillus* bacteria, three species of *Bifidobacterium* bacteria and *Streptococcus thermophilus*. The Multibiotic formulation contained a total of 21.075 billion Colony Forming Units (CFU) per capsule (Table 1).

**Table 1:** The study drug was a multi–species probiotic formulation containing seven bacterial species that was ARTG listed with the Therapeutic Goods Administration in Australia

PROBIOTIC species and strain identifier	CFU [live ells] / capsule
*Lactobacillus rhamnosus* [Med 26]	9 billion
*Lactobacillus acidophilus* [Med 27]	3.75 billion
*Lactobacillus plantarum* [Med 25]	1.575 billion
*Bifidobacterium animalis spp. lactis* [Med 13]	3 billion
*Bifidobacterium breve* [Med 12]	1.75 billion
*Bifidobacterium bifidum* [Med 11]	500 million
*Streptococcus thermophilus* [Med 51]	1.5 billion
Total	21.075 billion

*CFU*, Colony Forming Units / Capsule

The IP was manufactured in accordance with Good Laboratory Practice (GLP) and Good Manufacturing Practice (GMP) for toxicological and clinical studies, respectively. The intellectual property (IP) was produced utilising Medlab Clinical’s patent protected probiotic formulation. Manufacturing and validation of the IP was assigned under agreement to Nutribiotech Co Ltd., South Korea, whilst packaging and labelling was assigned to South Pack Laboratories Pty Ltd in NSW, Australia. Nutribiotech holds all relevant licences to manufacture in accordance with the Therapeutic Goods Administration (TGA) guidelines.

**Fig. 2:**
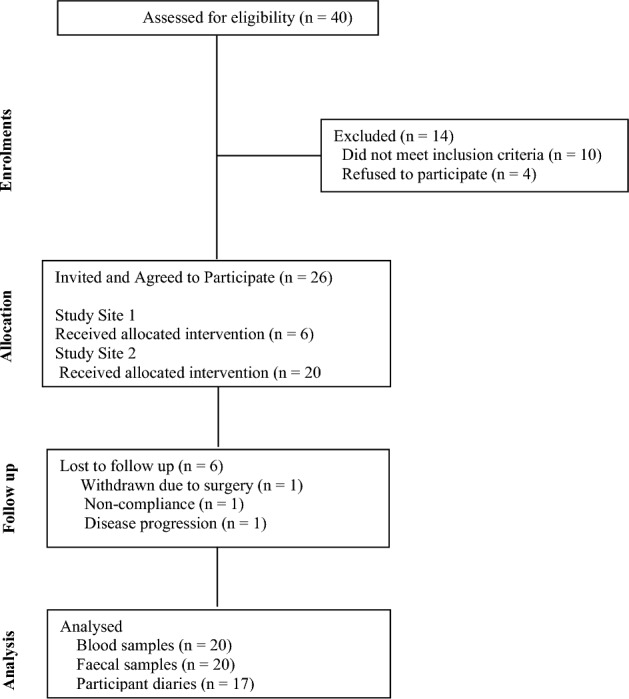
CONSORT Diagram of a single arm exploratory pilot study

The IP was supplied in a volume of 60 hard capsules in a sealed glass bottle. The average fill weight of each capsule was 280 mg and the average total capsule weight was 325 mg ± 7.5%. The IP presents as a hardshell capsule size 1 containing a fine off white speckled free-flowing powder. The IP was stored between 2°¾8° C away from direct heat and sunlight. The IP was packeged in 115 mL brown glass bottles with tamper proof lids, packaged into cartons of 10 bottles and shipped under ambient conditions to the clinical trial sites (Fig. 2).

### Participant inclusion and exclusion criteria

The inclusion criteria included (i) adult patients diagnosed with cancer and who were chemotherapy naïve or about to commence a new chemotherapy regimen due to disease progression; (ii) were aged 18 to 75 years; determined to have a life expectancy of more than 12 months; (iii) there was absence of any psychological, familial, sociological, or geographical conditions, which could potentially hamper compliance with the study protocol and follow-up schedule; (iv) participants agreed to comply with the study protocol; and agreed to participate following a signed and written informed consent.

Participant exclusion criteria included (i) active infections treated by antibiotic therapy; (ii) hypersensitivity to the study drug (i.e. Probiotics); (iii) any concurrent malignancy other than non-melanoma skin cancer; (iv) serious concomitant systemic disorders or diseases incompatible with the study; (v) patient has a history of primary psychological disorder; (vi) and illicit drug use.

### Faecal bacterial DNA extraction and sequencing

Stool samples were collected using a stool specimen collection kit. This collection kit included instruction for the stool sample collection, gloves, a sterile container, and sealed plastic pouch. Participants brought the stool sample to the clinic. Samples were stored at − 20 °C at the study centres before being transfer to the laboratory. DNA was obtained from human faecal material by using the QIAamp® DNA Stool Mini Kit (QIAGEN LTD. West Sussex, UK) and the Qiacube according to manufacturer’s guidelines. The samples of 200 mg were added to 1 ml InhibitEx buffer in labelled 2 ml microcentrifuge tubes stored in ice. Vortexed for 1 min and heated at 70 °C for 5 min. Then vortexed for 15 s before being centrifuged at 20,000 G/14,000 rpm for 1 min to pellet stool particles. 200 ml of supernatant aliquoted into the labelled microcentrifuge tubes. Whole-genome sequencing. The quantity and quality of the extracted DNA was determined using the Implen NanoPhotometer® (LabGear, Australia). DNA samples were stored at − 80 °C until analysis.

DNA was sequenced on the MiSeq 16S Metagenomics system using 300 bp paired reads and the MiSeq v3 reagents which sequences the V3 and V4 regions of the 16S rRNA gene (Illumina, Australia). Post-sequencing, Illumina’s BaseSpace metagenomics workflow was used to perform taxonomic classification based on the Greengenes database. This workflow demultiplexes indexed reads, generates FASTQ files, and then classifies reads at several taxonomic levels ranging from kingdom to species.

### Faecal microbial analysis

Due to COVID restrictions stool collection kit was mailed to participants. This collection kit includes instruction for the stool sample collection, gloves, a sterile container, and sealed plastic pouch. Participants were instructed to store the stool samples in their freezer at − 20° C and return it to the researchers soon after using a freezer bag. Samples were subsequently stored at ICON cancer centre at − 20° C.

Stool samples were collected from patients using the QIAamp® DNA Stool Mini Kit (QIAGEN Ltd. West Sussex, UK) and the Qiacube automated extractor according to the manufacturer’s guidelines. DNA extraction was be performed using the QIAamp DNA stool kit and faecal microbial composition was identified by sequencing the 16S rRNA using the Illumina next-generation sequencing system.

### Blood biochemistry

#### Short chain fatty acids

Blood samples were collected from 26 study participants following the method described. Twenty-six patients provided baseline samples. Twenty participants completed the study and provided a final blood sample for analysis.

A GC–MS method, with minor modifications from published methods, was employed to determine the level of SCFA in plasma samples (Garcia-Villalba et al. 2012; Juanola et al. 2019). A modified GC–MS method for analysis of SCFAs (i.e. acetic acid, propionic acid, butyric acid, iso-butyric acid and iso-valeric acid) using acetic acid-d4 stock solution as an internal standard was developed to provide a standard assay testing method for SCFAs in plasma collected from participants from two study centres. Quantitative and qualitative determination of SCFAs was performed on GC equipped with an automatic liquid sampler and coupled with an Agilent mass selective detector.

Analysis was performed using an Agilent 7890A gas chromatography system coupled to an Agilent 5975C inert XL EI/CI mass spectrometric detector (MSD, Agilent Technologies, Sydney, Australia). The column used was a high polarity, polyethylene glycol, fused silica capillary column Agilent J&W DB-WAXETR (30 m × 0.25 mm id × 0.25 μm film thickness).

Helium was used as the carrier gas at a flow rate of 1 mL/min. Injection was made in the spitless injection mode with an injection volume of 1 mL and an injector temperature of 250 °C. The column temperature was kept initially at 70 °C, increased to 100 °C at a rate of 10 °C/min and kept at 100 °C for 0.5 min increased to 130 °C at 6 °C/min and kept at 130 °C for 0.5 min increased to 180 °C at 10 °C/min and then increased to 220 °C at 40 °C/min., and kept at 220 °C for 1 min (total run time = 16 min). Solvent delay was 4 min.

The detector was operated in electron impact ionisation mode (electron energy 70 eV), scanning in the 30–250 m/z range. Mass spectral data was collected in SIM mode for quantification. Temperature of the ion source, quadrupole and interface was 230 °C, 130 °C and 250 °C respectively. Identification of acetic acid, acetic acid-d_4_, propionic acid, butyric acid, iso-butyric acid, and iso-valeric acid was based on the retention time of standard compounds and with the assistance of NIST MS library.

Identification of acetic, propionic, butyric, acids was based on the retention time of standard compounds and quantified using the Agilent Mass Hunter Quantitative version 8.07.00 software with the assistance of NIST MS search 2 libraries. Quantification of each SCFA was based on calibration curves obtained from increasing concentrations of standards diluted in ethyl acetate.

A characteristic single ion was selected for the quantification of each compound: acetic acid 60.1 m/z, acetic acid–d_4_ 63 m/z, propionic acid 74 m/z, iso-butyric acid 88 m/z, and butanoic acid 73 m/z and iso-valeric acid 87 m/z.

The use of extracted ion chromatograms (EICs) for area calculation and quantification reduced the possibility of misinterpreting overlapping peaks.

#### Lipopolysaccharides (LPS)

Metabolic endotoxemia was determined by measuring plasma LPS using a commercially available kit (Pierce™ Limulus amoebocyte lysate chromogenic endotoxin quantification kit from Thermo Fisher, Sydney, NSW, Australia) (Thermofisher-Scientific 2021). The LAL Chromogenic endotoxin quantitation kit measures the amount of endotoxin in a sample using the limulus amebocyte Lysate (LAL) assay. The endotoxin concentration is measured via a chromogenic (photometric) signal generated in the presence of endotoxins. The photometric signal is read by a microplate reader.

Analysis was performed in duplicate according to the kit manufacturer’s instructions. Six mL of blood was collected in EDTA-plasma tubes, immediately placed at 4 °C and then centrifuged for 15 min at 1000 × g (or 3000 rpm) at 2–8 °C.

Plasma samples were aliquoted in endotoxin-free tubes using endotoxin-free pipette tips and stored at –80ºC. Assay was performed in a prewarmed 96-well plate and maintained at 37 °C throughout the assay.

Plasma samples were diluted 50-fold in endotoxin-free water. Diluted samples, blank and endotoxin Standard dilutions (50 μL) were dispensed in each well. Freshly reconstituted Limulus Amebocyte Lysate (50 μL) was added to each well. The plate was shaken to aid mixing and incubated at 37 °C for 25 min.

Following incubation prewarmed (37 °C) chromogenic substrate (100 μL) was added to each well, and incubation was extended for an additional 6 min at 37 °C. The reaction was stopped by adding 25% glacial acetic acid (50 μl). Absorbance was measured using a 96-well plate reader at 405 nm (Thermo-Fisher-Scientific 2021).

#### Food frequency questionnaire

The EPIC-Norfolk Food Frequency Questionnaire was used to record and score nutritional intake during the cancer treatments as quintiles of fruit, vegetables and total fibre consumed during the study period, and values compared to previously published data (Bradbury et al. 2014). Due to COVID-restrictions the questionnaire was mailed to participants, and they were requested to return it via surface mail.

### Statistical analysis

Data were presented descriptive, as mean (95% CI) or median (95% CI) where appropriate. unless otherwise stated. Data were tested for normality of the distribution, and statistical analysis was performed with the statistical software STATA (version 17, Texas, USA) and GraphPad Prism 10 (California, USA).

**Fig. 3:**
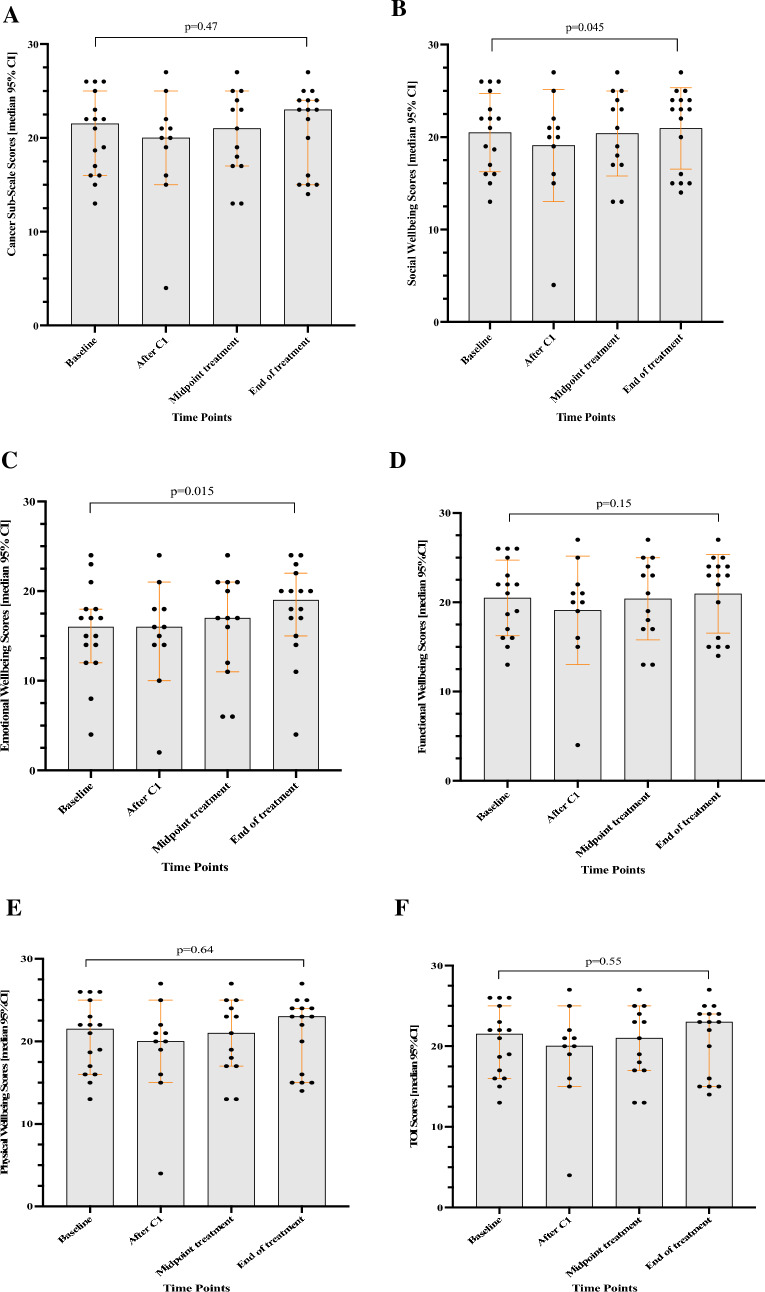
**(A–F)** Changes in Functional Assessment of Cancer Therapy–Colorectal (FACT C) scores across four time points

The Functional Assessment of Cancer Therapy–C (FACT–C) questionnaire was used to assess participants physical, emotional, social, and functional wellbeing. Comparison between the various FACT–C domains was carried out using the Wilcoxin signrank matched pairs test to determine positive or negative changes in scores at baseline and the end of treatment. The Z score and the *p*-significance indicated if there was a significant difference in median baseline vs. post-end of treatment rank scores (Table 3). Furthermore, the data were presented in graph form to show median scores changes with treatment progression [Graph Pad Prism (version 10.0)] (Figs. 3A–F).

The EPIC-Norfolk Food Frequency Questionnaire utilises the FFQ EPIC Tool for Analysis (FETA) to calculate nutrient and food group data from the entered food frequency questionnaires (FFQ). Excel table was further analysed by the FETA software application licenced under the General Public Licence, version 2 (GPL v.2).

Compositional changes in patients’ intestinal microbiomes were assessed by Linear Discriminate Analysis (LDA) effect size (LEfSE). The analysis found no significant differences after FDR corrections after a log LDA threshold cut-off of 2, Kruskal–Wallis *p* value < 0.05 for any taxon. Adonis function in R (version 3.2) was used for the statistical analysis of the relative abundances of bacterial genera beta-diversity as calculated by Brady–Curtis dissimilarities. Principal Coordinates Analysis (PCoA) was graphically shown using a 2D distribution. Permutational Multivariate Analysis of Variance (PERMANOVA) was carried out at the phyla and species levels.

## Results

Demographical characteristics and treatment regimens of the participants that were enrolled in the study are presented in Table 2. Participants dropout was mainly due to the COVID restrictions that had been imposed. The dropout rate was 23% (*n* = 6) throughout the study.

**Table 2:** Respondent demographical characteristics and treatment regimens

ID	Age (sex)	Weight Kg	Cancer site	Treatment regimen
1	60 (M)	79	Colon	FOLFOX, 5-FU bolus. number of cycles: 12 cycles
2	77 (F)	82	Colorectal	FOLFIRI + Bevacizamab
3	70 (F)	75	Colorectal Liver	FOLFOX + Avastin then ZUCERO study (PG545 + Nivoluimab)
4	44 (M)	67	Rectum	FOLFIRI
5	72 (F)	53	Colorectal	FOLFIRI
6	39 (F)	52	Rectum	FOLFOX; Capecitabine; FOLFIEI + Avastin
7	39 (M)	85	Colon	Previously Fluorouracil–irinotecan–leucovorin (FOLFIRI) followed by Bevacizumab–Capectitabine. Fluorouracil–leucovoin–oxaliplatin (modified FOLFOX 6)
8	58 (F)	74	Colon	Capecitabine–OXALIplatin (XELOX) 1 cycle, following by Fluorouracil-Leucovorin–OXALIplatin (modified FOLFOX 6) nine cycles, completed seven cycles during probiotic trial, last two cycles not completed
9	65 (F)	*	Rectum	Capecitabine four cycles—plus Radiotherapy
10	66 (F)	90	Colon	Bevacizumab–Fluorouracil–irinotecan–leucovorin (FOLFIRI + Bevacizumab), two cycles. Bevacizumab–Capectitabine, three cycles. Radiotherapy
11	70 (F)	*	Lung	FOLFIRI
12	58 (F)	79	Breast	Pertuzumab, trastuzumab and taxol followed by PACLitaxel–PERtuzumab-TRAStuzumab with DENOsumab five cycles before starting probiotic
13	65 (F)	59	Pancreas	Gemcitabine–PAClitaxel (nanoparticle albumin bound) 3 out of 4 weeks. Four cycles
14	77 (F)	63	Breast	Paclitaxel–Trastuzumab, four cycles
15	62 (F)	66	Breast	Cyclophosphamide-doxorubicin (AC dose dense2 weekly), two cycles. Paclitaxel–Trastuzumab three cycles
16	72 (F)	63	Oesophagus	Cisplatin–fluorocuracil (4 day infusion). Followed by Cisplatin–fluorouracil and radiotherapy, three cycles
17	46 (F)	64	Breast	Goserelin monthly (three of six cycles), Cyclophosphamide-doxorubicin (AC dose dense 2 weekly) four of eight cycles, Paclitaxel monthly, three cycles
18	63 (F)	*	Breast	Cyclophosphamide–doxorubicin (AC dose dense 2 weekly). Paclitaxel weekly (3 months)
20	43 (F)	48	Breast	Gsoerelin monthly (four cycles), Cyclophosphamide–Doxorubicin AC dose dense 2 weekly, Paclitaxel three cycles
22	55 (F)	79	Breast	Cyclophosphamide–doxorubicin AC dose dense 2 weekly (four cycles). Paclitaxel (three cycles)
23	66 (F)	79	Breast	Cyclophosphamide-doxorubicin AC 3 weekly (four cycles) Paclitaxel three cycles
24	69 (M)	*	Oesophagus	Pembrolizumab 3 weekly flat dose (four cycles)
25	F	*	Rectum	Cetuximab–flourouracil–irinotecan–leucovorin
26	F	*	Breast	Everolimus–Exemestane

* Missing data; *FOLFOX* Combination Folinic acid Fluorouracil Oxaliplatin; *Combination FOLFIRI* Folinic acid Fluorouracil Irinotican]

As shown in Table 2 there was a heterogeneity of diagnosed cancers that resulted in participants receiving a wide variety of treatments including chemotherapy, targeted therapies, immunotherapy, radiotherapy, and surgery during the study. It was initially expected that participants would be followed for 3–cycles of a given chemotherapy regimen, notwithstanding enrolled participants had complex treatment regimens which were frequently adjusted or changed. Hence, time spent on the study varied from 4 to 32 weeks with a median duration of 15 weeks.

### Endotoxemia

Twenty patients provided at least two blood samples. The plasma level of endotoxin was less than 1.0 EU/mL in all patients except in one patient whose level increased from 0.302 at baseline to 1.763 EU/mL at the final time point. The mean (SD) plasma level of endotoxin was 0.309 ± 0.195 at baseline compared to 0.350 ± 0.0.335 EU/mL at the final time point. Several factors may have influenced this result.

Despite using the low range concentration curve (0.01 to 0.1 EU/mL), undetected LPS was observed even for spiked samples, suggesting that plasma samples may have components that produce inhibitory effects of the kit reaction. Literature reports have suggested that the interfering substances may originate from plasma or from the anticoagulant used for plasma preparation (e.g. EDTA).

### Participant wellbeing

Effect of Probiotic Treatment on Participant Wellbeing | FACT–C (primary endpoint). The FACIT with subscale C which included additional questions related to the digestive system was used as the present study sought to evaluate the impact of a probiotic intervention on digestive and intestinal health. Sixteen of 26 patients who consented to participate in the study filled out the FACT C questionnaire. The results are graphed as a scatter plot (Figs. 3 A–F) and tabulated in Table 3. The Trial Outcome Index (TOI) was the sum of the Physical Well-Being (PWB), Functional Well-Being (FWB), and the FACT–C subscale. The Trial Outcome Index (TOI) is not a standalone FACIT measure. It is the sum of the Physical Well-Being (PWB), Functional Well-Being (FWB), and FACT C subscale. The TOI is an efficient summary index of physical/functional outcomes. It is a common endpoint used in clinical trials because it is responsive to change in physical/functional outcomes, sometimes more than a total (overall) multidimensional aggregated score which includes social and emotional wellbeing (Cella et al. 1993).

**Table 3:** FACT C mixed-effects analysis comparing baseline vs. end of treatment

	Comparison (*n*) observations	Wilcoxon signed-rank test for matched pairs	*p* value
CSS*	Baseline (16) vs. end of treatment (16)	z = − 0.73 | Prob >|z|= 0.467	0.47
SWB	Baseline (16) vs. end of treatment (16)	z = 1.95 | Prob >|z|= 0.051	0.055
EWB	Baseline (16) vs. end of treatment (16)	z = − 2.43 | Prob >|z|= 0.015	0.015
FWB	Baseline (16) vs. end of treatment (16)	z = − 1.48 | Prob >|z|= 0.140	0.15
PWB	Baseline (16) vs. end of treatment (16)	z = 0.467 | Prob >|z|= 0.640	0.64
TOI	Baseline (16) vs. end of treatment (16)	z = 0.600 | Prob >|z|= 0.551	0.55

*CSS Cancer sub-scale; *SWB* Social wellbeing; *EWB* Emotional wellbeing; *FWB* Functional wellbeing; *PWB* Physical wellbeing; *TOI* Trial Outcome Index

It was noted that all domains of the FACT C improved from baseline to end of treatment albeit not all significantly (Figs. 3 A–F). Social wellbeing scores (borderline significant at the end of treatment *p* = 0.045) and physical wellbeing scores (not significant at the end of treatment *p* = 0.64) reduced from baseline to after the first round of chemotherapy but increased to above baseline levels at the end of the study treatment. Emotional wellbeing increased from baseline to the end of the study treatment with significant improvement (*p* = 0.015) and the cancer subscale, used to measure changes in bowel habits and function, were similar at all time points (Table 3; Fig. 3A) with no significant improvement (*p* = 0.47).

### EPIC food frequency questionnaire

The cohort of cancer patients in the present study had a high intake of fruits, vegetables, and fibre. The average fruit consumption was 285 g per day (4th quintile) directly comparative to the intake in the 4th highest quintile reported in the Bradbury review (Bradbury et al. 2014). The vegetable intake was 449 g per day (5th quintile) and the fibre intake was 24 g per day (4th quintile). Accordingly, the cohort of cancer patients in the present study had a high intake of fruits, vegetables, and fibre during their cancer treatments.

### Bowel function effects

Participants were asked to record the Bristol stool scores on three consecutive days over three time points during the study. Twenty participants returned diaries with Bristol Stool scores. Additional information relative to stool consistency and frequency was obtained from access to medical records. Participants experienced the full range of stool consistency and frequency scoring from 1 to 7 on the Bristol scale.

Three participants reported improvement in constipation to a level where Movicol or other medication was ceased. However, one participant reported worsening of constipation whilst administering the probiotics, which did not improve upon ceasing the multi-biotic probiotic test formula.

Two participants had relief of chronic diarrhoea, one reporting normal stool for the first time in 12 months. The other reported alternating diarrhoea and constipation that was improved whilst administering the multi-strain probiotic test formula. Of the two participants with a stoma, one reported no difference whilst the other experienced significant relief from painful constipation. Five participants reported no noticeable effects. Eight participants maintained normal stool during the treatment period and reported no symptoms of significant bowel toxicity. Overall, 85% of participants reported improvement in constipation and or diarrhoea or absence of significant disturbance to bowel function.

From those patients with self-reported intestinal symptoms during the study, five participants had normalised bowel function, one had worsened constipation and the thirteen other participants reported maintenance of normal bowel function throughout the study period. That is 85% of participants reported improvement in constipation and or diarrhoea or reported the absence of any significant abdominal disturbances to bowel function during the study. Specifically, in patients with colorectal cancers (n = 6) administration of a multi-strain probiotic formulation improved bowel symptoms and quality of life.

### Chemotherapy-induced mucositis effects

Two participants developed bowel toxicity during the trial period. One participant developed inflammation of the terminal ileum (CTCAE: Grade 2, moderate ileitis) lasting five days. This occurred just 2 weeks after commencing the study medication (i.e. multi-strain probiotic). The consulting oncologist reported that the adverse event was unlikely to be related to the study medication. No action was taken in relation to ceasing the multi-strain probiotic. A second participant developed CTCAE Grade 2 toxicity in response to Taxol. This participant experienced very high levels of stress and anxiety for which Valium was prescribed. In this participant chemotherapy was causal for diarrhoea and cramping which improved with a reduction in Taxol dosage. The participant continued to report alternative diarrhoea with constipation. Bowel irritation was reported reduced, nevertheless, this participant continued to experience reflux and nausea, eventually prescribed regular use of metoclopramide. Bowel motions continued to be frequent (3–4 times per day) and soft (i.e. due to prokinetic action of Maxolon). One participant (i.e. ID#6) developed pneumonia and was reported as severe, and unlikely related to study drug. The patient recovered following prescribed intravenous antibiotics.

### White blood cell and neutrophil counts

**Fig. 4:**
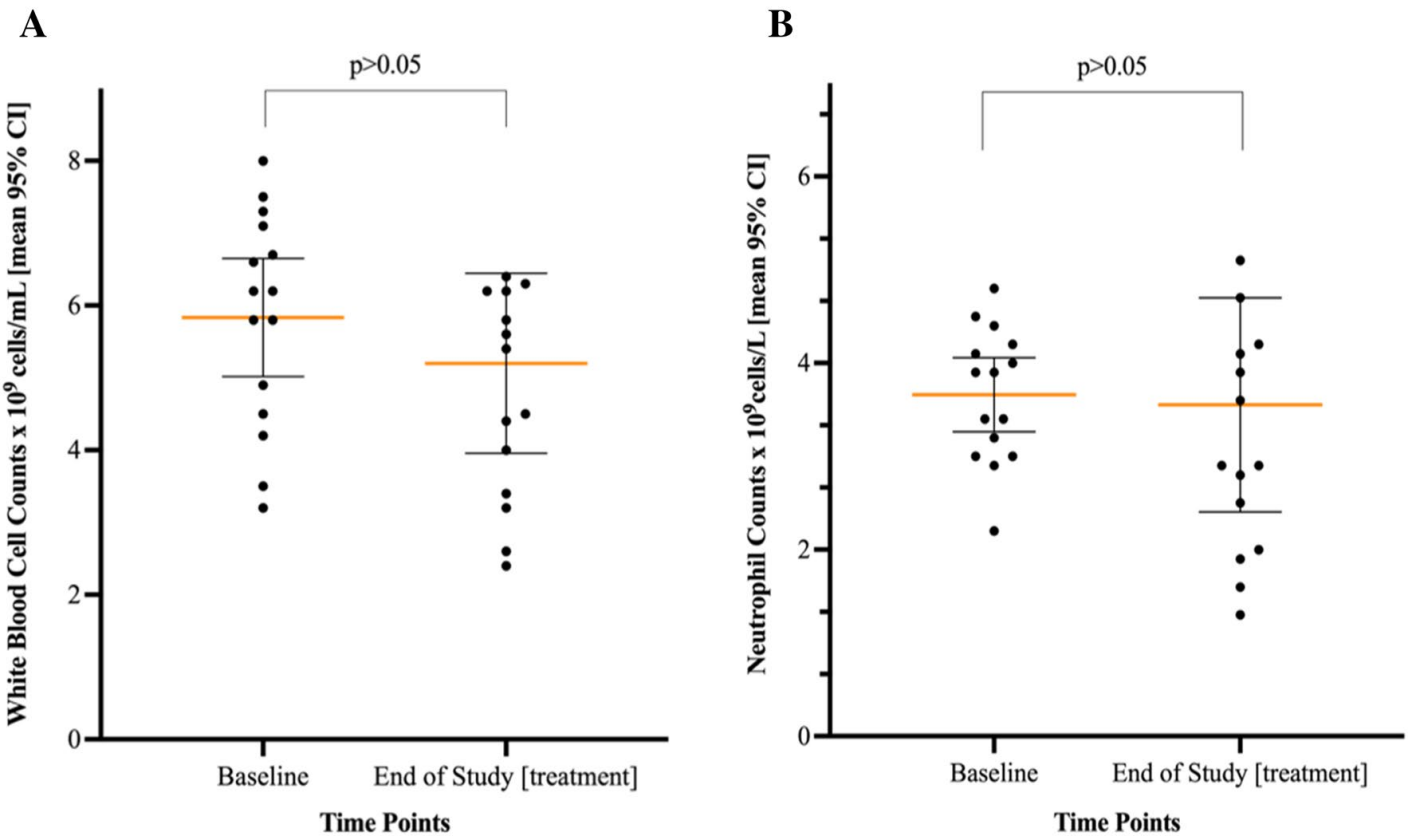
**A, B** Scatterplot of white blood cells and neutrophil counts from baseline compared with end of study (treatment). Each dot-plot represents one study participant scattered around the mean (horizontal line)

Participants administered the investigational product over a variable amount of time namely from 4 to 32–weeks with a median duration of 15 weeks (IQR = 10–20.5). The white blood cells and neutrophils were measured (Figs. 4A, B) at two timepoints. Figure 3 presents a graphical representation of the changes in white blood cell and neutrophil counts from baseline to the end of the treatment period.

A pairwise comparison result post-hoc test, showed that there was no significant difference (*p* > 0.05) in white blood cell counts from the 2-time points, namely at baseline and at the end of study treatment period with mean (95%CI) of 87.5 (75.2–99.8) and 78.0 (59.3–96.7), respectively. Furthermore, the analysis showed that there was no statistically significant difference (*p* > 0.05) between the 2-time points in blood neutrophil counts at baseline and at the end of study treatment with mean (95% CI) of 54.9 (48.0–60.9) and 53.3 (36.1–70.5), respectively with an F (0.83) and *p* = 0.37.

A clinical results report showed that only one participant developed febrile neutropenia. This participant was first diagnosed with a 39 mm mid-low rectal carcinoma, seen on colonoscopy for rectal bleeding. Hepatic metastases on diagnosis. Induction FOLFOX for six cycles. Three months of chemotherapy (FOLFOX) prior to starting multi-strain probiotic trial. Regimen changed to FOLFIRI plus Avastin. Two weeks later, hospitalised due to pneumonia, fever, CRP 100 and diagnosed with febrile neutropenia. Resumed probiotics two weeks later and completed the probiotic intervention ten weeks later with no further complications.

### Intestinal microbiome analysis

#### Phylum level changes

**Fig. 5:**
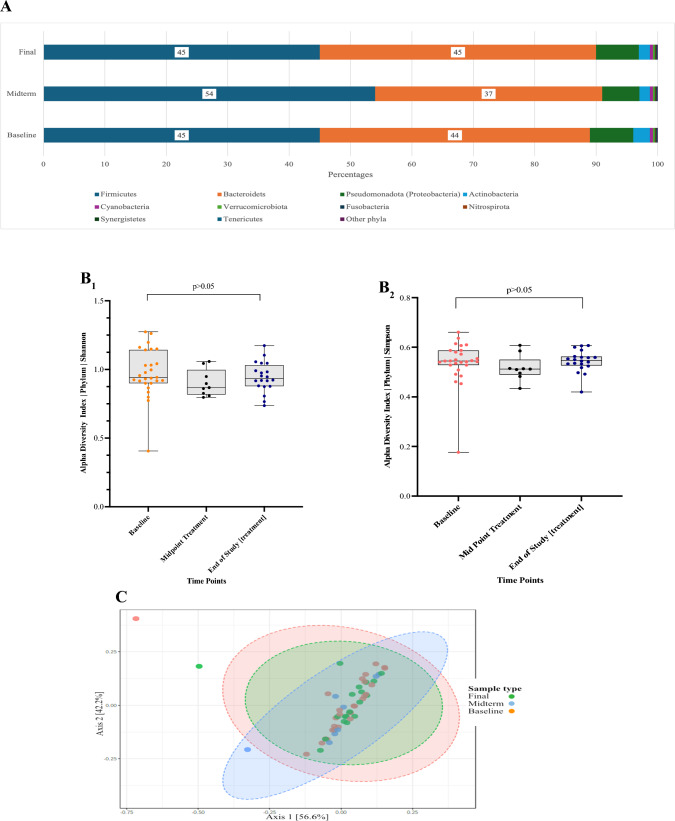
**A** Phylum-level alpha diversity. The Shannon and Simpson Diversity Index (species richness) showed no significant differences in the phyla alpha diversity at different timepoints namely, Baseline, Midpoint treatment and End of treatment sample conditions*.* Changes in relative abundance at the phylum level. Baseline, Midpoint treatment, End of Study treatment. Difference in alpha diversity at the Phylum level are presented in panel (5B_1_) Shannon Index *p* value: 0.22008; Kruskal–Wallis, and in panel (**5B**_**2**_) Simpson Index, *p* value: 0.17621; Kruskal–Wallis. **C** The beta-diversity Bray–Curtis’s distance matrix analysis for the faecal samples. No changes in beta-diversity were observed at the phylum level (PERMANOVA, [*F*–value: 0.53842; R-squared: 0.019913; *p* < 0.768])

Of the enrolled 26 adult participants, 4 participants withdrew due to disease progression and did not take the probiotic test medication. Twenty participants provided a stool sample at base line and at the end of study treatment. Eight participants also provided a midpoint (i.e. mid-treatment) faecal sample. The data was analysed for changes over time in terms of the relative abundance and diversity in the intestinal microbiome at the phyla levels (Fig. 5).

**Fig. 6:**
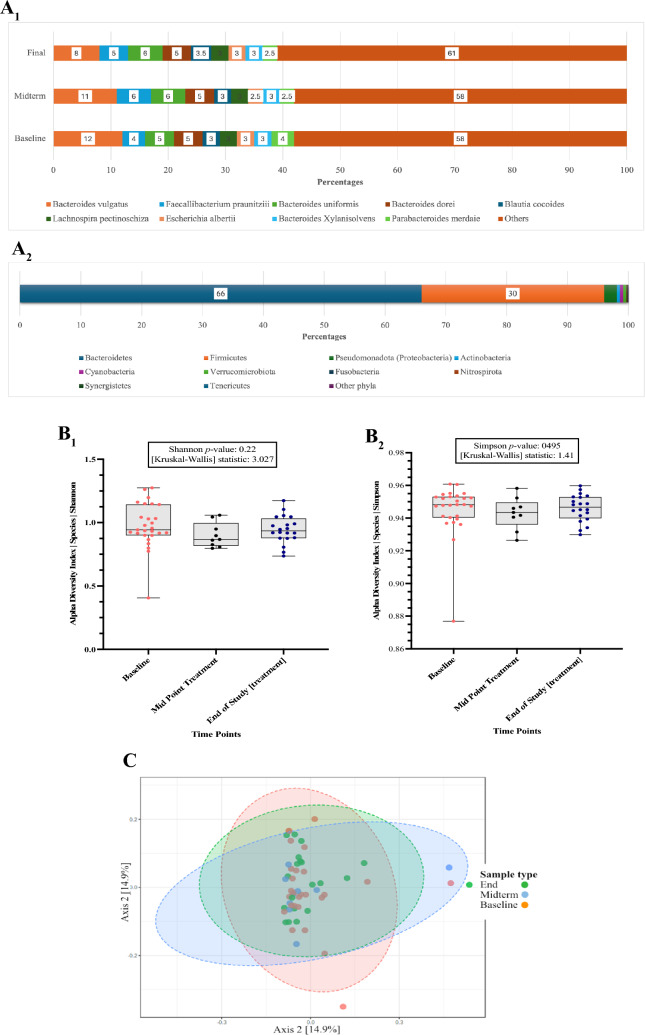
Changes in relative abundance at the species **A**_**1**_ and phyla **A**_**2**_ level at Baseline, Midpoint treatment, End of Study treatment. Difference in alpha diversity at the species level by **6B**_**1**_)Shannon and **6B**_**2**_ Simpson Index. **6C** The beta-diversity Bray–Curtis’s distance matrix analysis for the faecal samples. The comparison performed with the Principal Coordinates Analysis (PCoA) is shown using a 2D distribution. Permutational Multivariate Analysis of Variance (PERMANOVA). [*F*–value: 0.40783; R-squared: 0.015156; *p* < 0.994]) was used for statistical significance analysis

The data was analysed for changes over time in relative abundance and diversity of the intestinal microbiota at the species level (Fig. 6).

The longitudinal variation in relative abundance — the fold-change (log_2_ fold-change) from baseline to final time point of the microbial taxa was examined. Only one species was found to show a significant change in relative abundance. *Bacteroides xylanisolvens* was found to have decreased 1.3-fold from baseline to final time point (*p* = 0.034) difference in fold change. *Bacteroides ovatus* and *Bacteroides xylanisolvens*, were shown in a study positively correlated with treatment outcomes in a heterogeneous cohort that included multiple cancer types. The bacteria were significantly enriched in responders compared to non-responders in the study. Oral gavage of these bacteria enhanced the efficacy of erlotinib in a murine lung-cancer model. This study also found that *Bacteroidetes* (44.51%) and *Firmicutes* (44.04%) were the dominant phyla accounting for nearly 90% of the intestinal microbiome and present in equal proportions (Heshiki et al. 2020). Twenty-nine species had a fold change greater than (*n* = 12) or less than (*n* = 17) than 2 as shown in Table 4.

**Table 4:** Log_2_ changes greater than or less than a twofold change

Taxa	Fold change
*Enterococcus durans*	− 19
*Clostridium perfringens*	− 16
*Enterococcus lactis*	− 12
*Clostridium butyricum*	− 9
*Clostridium saccharobutylicum*	− 6.4
*Clostridium neonatale*	− 3.2
*Odoribacter laneus*	− 3
*Prevotella loescheii*	− 3
*Klebsiella pneumoniae*	− 2.5
*Bifidobacterium bifidum*	− 2.2
*Bacteroides fragilis*	− 2.2
*Enterobacter amnigenus*	− 2
*Bacteroides salanitronis*	2.02
*Prevotella paludivivens*	2.04
*Candidatus liberibacter africanus*	2.06
*Dialister invisus*	2.25
*Proteus penneri*	2.33
*Paraprevotella xylaniphila*	2.39
*Campylobacter gracilis*	2.44
*Prevotella albensis*	3.98
*Propionigenium modestum*	4.1
*Bacteroides intestinalis*	4.34
*Cetobacterium ceti*	4.61
*Megasphaera micronuciformis*	5.6
*Lactobacillus taiwanensis*	5.92
*Lactobacillus gasseri*	6.48
*Enterobacter ludwigii*	11.6
*Ruminococcus flavefaciens*	12.91
*Prevotella buccae*	20.15

#### Core microbiota unique species at baseline vs. end of treatment

Fifteen species were found to be uniquely present at baseline or at the end of study final time point. *Propionigenium modestum, Enterobacter soli. Parabacteroides gordonii, Eubacterium cylindroides, Megasphaera micronuciformis, Escherichia coli* and *Prevotella buccae* were present at baseline but not at the final time point. *Clostridium perfringens, Prevotella maculosa, Clostridium neonatale, Fusobacterium gonidiaformans, Bifidobacterium bifidum, Dysgonomonas capnocytophagoides, Paraprevotella xylaniphila* and *Klebsiella variicola* were present at the final time point but absent at baseline (Table 5).

**Table 5:** Unique species at baseline and at the end of treatment time point

TAXA	B.abu*	F.abu	B.occ	F.occ
Baseline				
* Propionigenium modestum*	0.051	0.012	0.48	0.30
* Enterobacter soli*	0.051	0.040	0.44	0.35
* Parabacteroides gordonii*	0.053	0.058	0.40	0.35
* Eubacterium cylindroides*	0.078	0.066	0.44	0.35
* Megasphaera micronuciformis*	0.106	0.019	0.40	0.35
* Escherichia coli*	0.107	0.070	0.64	0.30
* Prevotella buccae*	0.262	0.013	0.44	0.15
End of study				
* Clostridium perfringens*	0.007	0.110	0.16	0.55
* Prevotella maculosa*	0.045	0.047	0.32	0.45
* Clostridium neonatale*	0.05	0.162	0.20	0.40
* Fusobacterium gonidiaformans*	0.055	0.056	0.32	0.40
* Bifidobacterium bifidum*	0.055	0.122	0.32	0.60
* Dysgonomonas capnocytophagoides*	0.073	0.104	0.32	0.45
* Paraprevotella xylaniphila*	0.088	0.037	0.36	0.55
* Klebsiella variicola*	0.114	0.146	0.36	0.50

* *B.abu* baseline abundance; *F.abu* end of treatment abundance; *B.occ* Baseline occurrence; *F.occ* end of treatment occurrence

### Individual patient observations

Levels of *Clostridium cadaveris* was found to be very high at the mid-term test during which time the patient experienced watery diarrhoea. A participant with a permanent stoma post colostomy following a diagnosis of colorectal cancer had experienced watery diarrhoea for a 2-month period upon entry to the present study. The participant noticed rapid improvement and the diarrhoea was resolved with continued probiotic administration.

### Comparison with data from the human microbiome project

The average abundance data from the stool samples of the participants in the present study were compared to the published data set of healthy volunteers of the Human Microbiome Project (HMP) (Consortium 2012).

The data set from the HMP contains 224 samples from medically assessed healthy volunteers. The stool samples were sequenced by 16S rRNA and the average abundance was calculated for each taxon down to genus level and compared with the microbiome of the participants in the present study at baseline to examine the relative differences between a healthy cohort and a cohort of cancer patients undergoing treatment. The sample size (*n* = 20) from the present study is smaller compared to HMP (*n* = 242), however the HMP data set are from healthy controls, using all samples in the HMP as controls is justified to reduce the impact of the high variability of the samples.

**Fig. 7:**
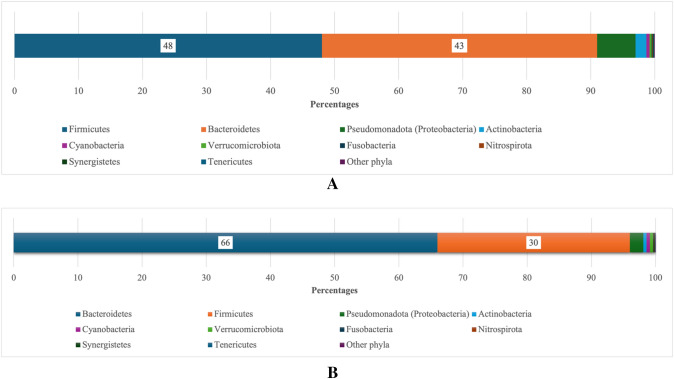
**A** HMP: Nine most abundant phyla, Microbiome Analysis. **B** Mucositis study: Application Microbiome Analyst. Phyla < 10 have been merged. Application Microbiome Analyst

Analysis at the phyla level using the online application Microbiome Analyst (Chong et al. 2020; Dhariwal et al. 2017). Identified 28 phyla with counts from the two datasets. Filtered by a minimum count of 4, low variance filter set to 10%, identified nine phyla as described in Figs. 7A, B.

**Fig. 8:**
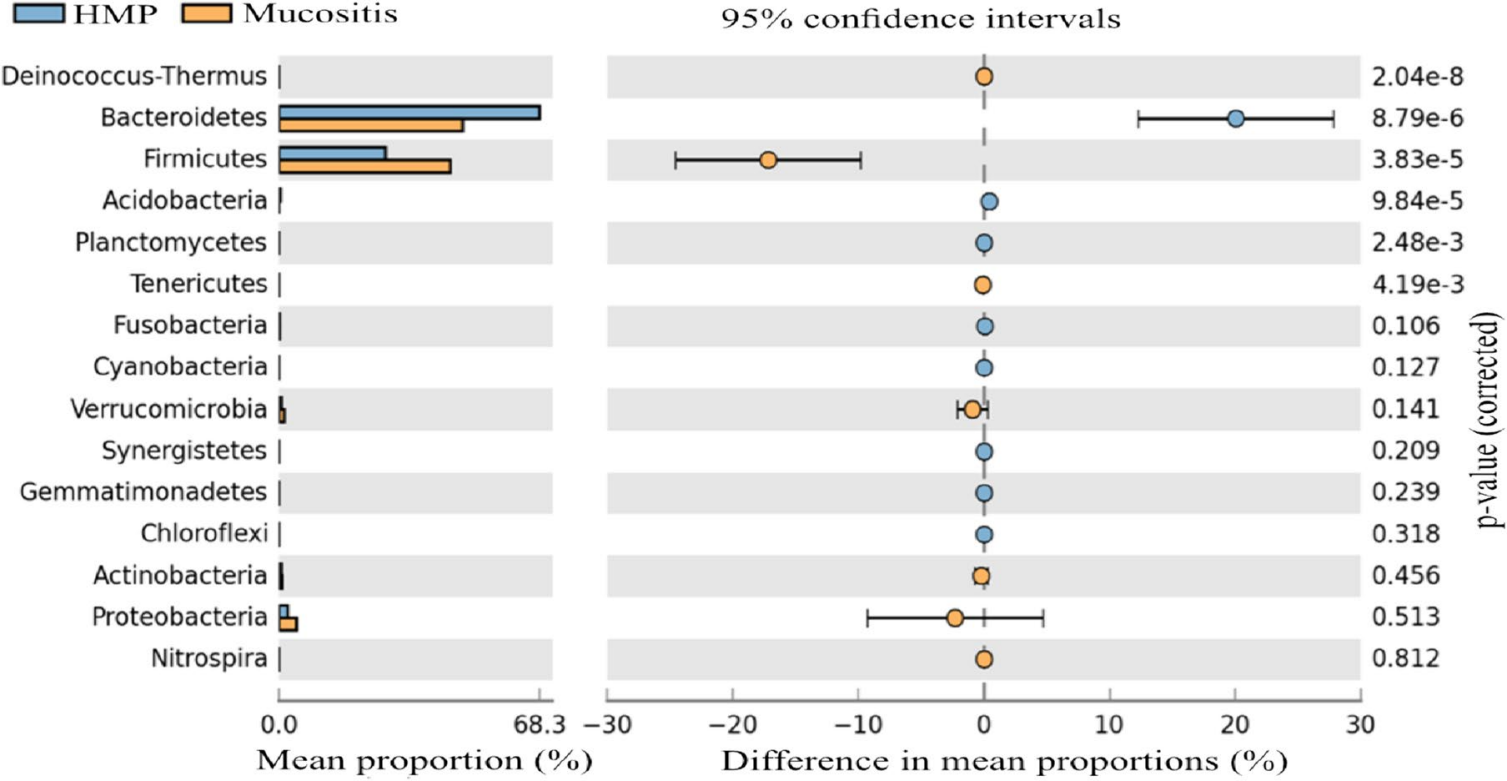
Extended error bars, phyla level, STAMP software

The data was also analysed using the online application, The Statistical Analysis of Taxonomic and Functional Profiles (STAMP) graphical software package, using the statistical G-test with Yeats plus Fisher’s (Type: Two-sided, CI Method: Asymptotic-CC, 0.95%) (Parks et al. 2014). The analyses found that Firmicute phyla is significantly more abundant and *Bacteroidetes* significantly less abundant in the cancer patients in the participants in present study compared to the healthy volunteers in the Human Microbiome Project (Fig. 8).

**Fig. 9:**
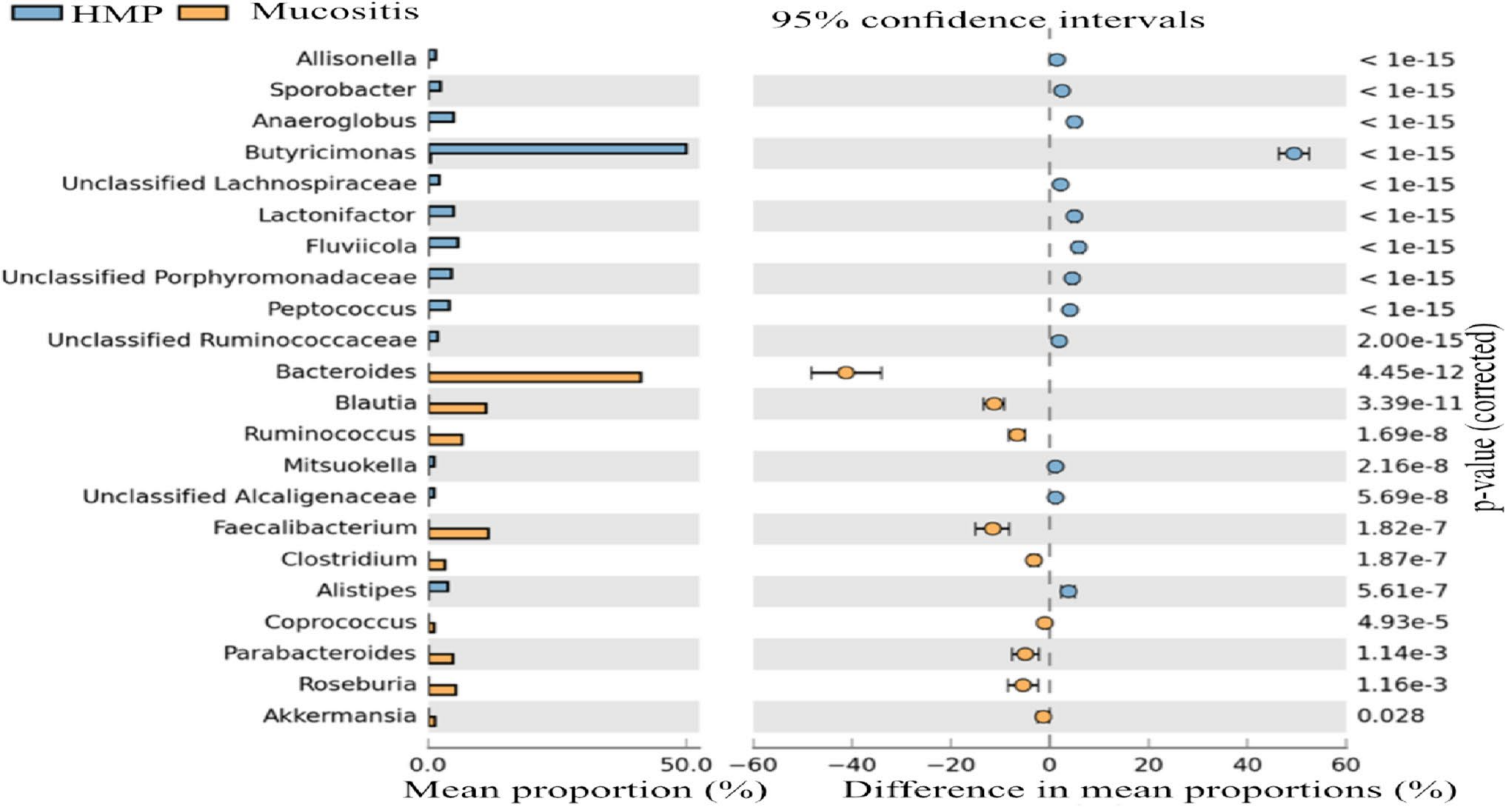
Extended error bars, genus level, STAMP software

Genera with an average abundance of above 0.01% selected from both datasets and genera with no hits in either were excluded. The data was analysed using the online, The Statistical Analysis of Taxonomic and Functional Profiles (STAMP) graphical software package, using the statistical G-test with Yeats plus Fisher’s (Type: Two-sided, CI Method: Asymptotic-CC, 0.95%) (Parks et al. 2014). The analyses found that the genera *Allisonella, Sporobacter, Anaeroglobus, Butyricimonas, Lactonifactor, Fluviicola, Porphymonadaceae, Peptococcus and Ruminococcaeae* were significantly more abundant in the HMP cohort whilst *Bacteroides, Blautia, Ruminococcus, Faecalibacterium, Clostridium, Parabacteroides, Roseburia and Akkermansia* were significantly more abundant in the participants in present study compared to the healthy volunteers in the Human Microbiome Project (Fig. 9).

### An exploratory study of intestinal microbiome patterns in exceptional responders to anticancer drugs

This part of the study recruited 14 patients (Table 6) who had been diagnosed with cancers and were deemed to be in remission for many years. Seven of their partners also agreed to participate in the study (seven females). The average age was 75 years with a range of 65.8–87.2 years. The average survival (SD) time was 78 months (6.5 years) with a range of 51–132 months.

**Table 6:** Exceptional survivors with cancer cohort

Sex	Age at end of study	Cancer site	Months survival since initial diagnosis
M	67.9	Oesophagus	72
F	72.9	Colon	74
M	87.2	Colon	63
M	72.8	Rectum	132
M	85.2	Melanoma	86
M	78.9	Melanoma	76
M	66.8	Rectum	78
F	65.8	Rectum	79
M	79.0	Stomach	92
M	73.5	Melanoma	51
M	68.5	Melanoma	67
F	72.3	Colon	58
M	80.9	Melanoma	58
M	75.8	Lung	102

### Intestinal microbiota analysis

**Fig. 10:**
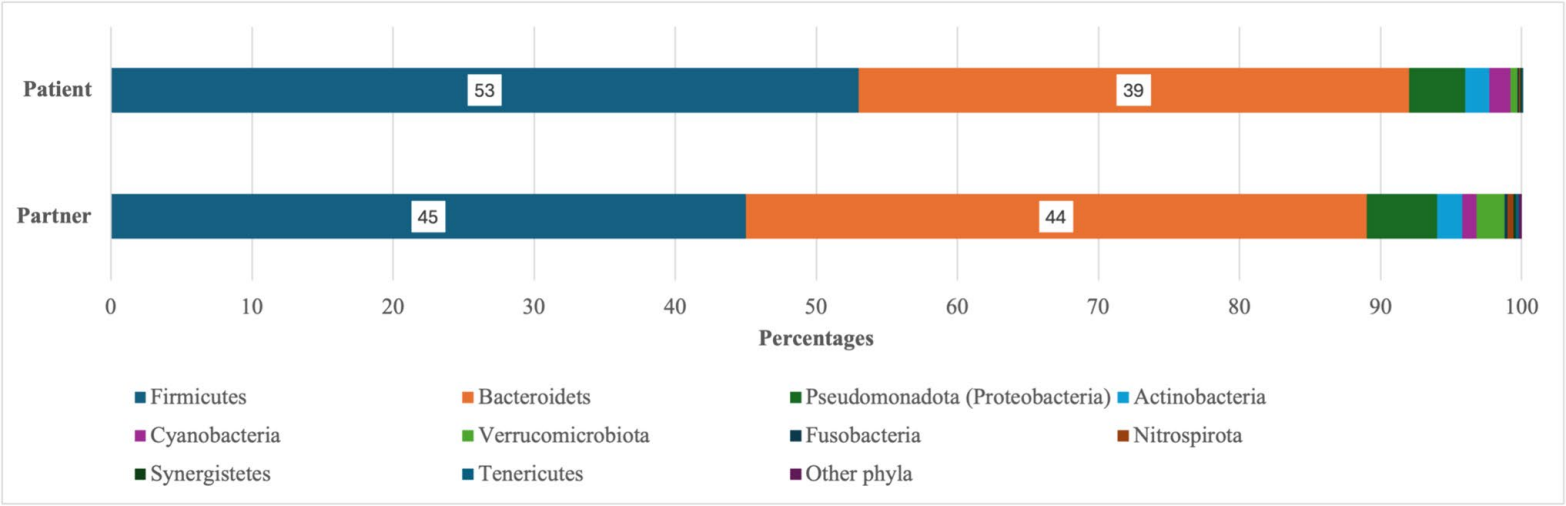
Percentage abundance at the phyla level, responder patients vs. partners. Responder patients had a similar relative (percentage) abundance at the phylum level to the partner cohort

**Fig. 11:**
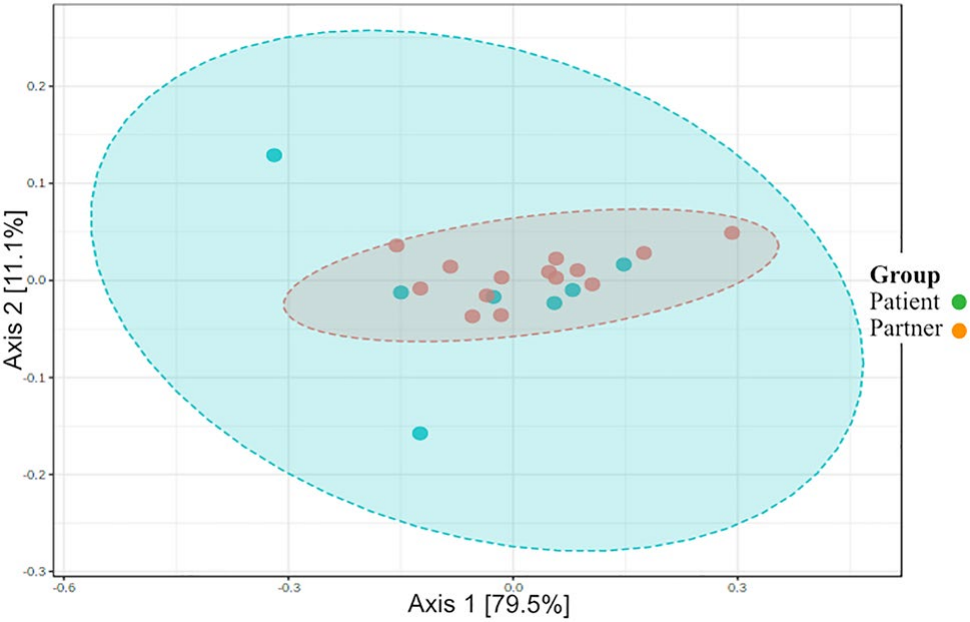
Beta diversity, phyla level, responder study vs. the partner patient cohorts. The differences are not significant [PERMANOVA] *F*-value: 1.1474; R-squared: 0.056952; *p* = 0.293

Responder patients had a similar relative (percentage) abundance at the phylum level to the partner cohort (Fig. 10). The differences are not significant [PERMANOVA] F-value: 1.1474; R-squared: 0.056952; *p* = 0.293 (Fig. 11).

## Discussion

This pilot clinical study was an exploratory supportive care investigation. The study was conducted at the height of the Covid-19 pandemic. Consequently, there was a participation loss of 23% throughout the study, a much lower participant loss than was anticipated. The attrition rate in clinical studies with cancer patients has been reported to be 33%, with variation amongst countries (i.e. range of 22%—39%) (Perez-Cruz et al. 2018). Various reasons can account for clinical trial participant dropouts, and these can include increased physical symptoms, poorer performance status, cognitive failure, and shorter duration of survival from cancer diagnosis and treatment related toxicities (Perez-Cruz et al. 2018). In this pilot study COVID restrictions were the predominant reason for participant dropout. The participants that returned food frequency questionnaires and that remained in the study, recorded a high consumption of fibre rich foods that may have further improved bowel function throughout the study.

This exploratory study confirmed that the administration of a multi–strain probiotic formulation was safe and tolerable in patients diagnosed with cancer who were undergoing various treatments for cancer. Radiotherapy and chemotherapy with surgery are critical components of cancer treatments. Consequently, approximately 90% of patients undergoing pelvic radiotherapy (Wang et al. 2015; Liu et al. 2021) or with intravenous chemotherapy (González-Mercado et al. 2020) will show gastrointestinal toxicity with gastritis and bloody diarrhoea, indicative of intestinal dysbiosis with the depletion of some keystone intestinal bacterial species such as those from the *Bifidobacterium, Lactobacillus* and *Enterobacteria* (e.g. *E coli*) families (Montassier et al. 2015). We previously reviewed the adjunctive use of compounds and probiotics administered to treat and prevent chemotherapy and radiotherapy induced intestinal mucositis (Thomsen and Vitetta 2018) and concluded that probiotics had a good safety profile and consistent with the posit in this study that a multi-strain probiotic formulation could reduce the severity of cancer-treatment side effects that can ensue.

Furthermore, in those patients that provided stool samples, at baseline there was observed a 1:1 ratio of *Firmicutes* to *Bacteroidetes*. During treatment with a multi–strain probiotic the *Firmicutes* to *Bacteroidetes* ratio increased and then returned to baseline by the end of the study. A decrease of 1.3–fold in abundance of the bacterium *Bacteroides xylanisolvens* from baseline to end of study treatment was observed. The administration of a multi–strain probiotic with *Bifidobacterium* and *Lactobacillus* species could have affected *B. xylanisolvens* levels reducing xylan fermentation and the production of the gut elaborated SCFAs acetate, succinate, and propionate (Chen et al. 2020). It is plausible that SCFAs produced by probiotic species provided alternative sources of SCFAs (Vitetta et al. 2023) for the cross-feeding actions that occur in the colon between acetate and propionate producers such as occurs from *Bifidobacterium* species which in turn provide bifidogenic energy sources for butyrogenic bacteria from the *Bacteroides* genus that can then improve barrier function of colonic epithelial tight junctions scaffolds.

Albeit not significant, over the time–course of the study *Enterococcus durans, Clostridium perfringens, Enterococcus lactis, Clostridium butyricum,* and *Clostridium saccharobutylicum* decreased more than sixfold; and nine species namely *Propionigenium modestum, Bacteroides intestinalis, Cetobacterium ceti, Megasphaera micronuciformis, Lactobacillus taiwanensis, Lactobacillus gasseri, Enterobacter ludwigii, Ruminococcus flavefaciens* and *Prevotella buccae* increased at least sixfold.

The main limitation of this study reflected the small heterogeneous sample of patients and treatments that participated in the study during a post infection time with Covid-19. Furthermore, the immune regulation and gut improvement effects that were implied and observed (e.g. decreases in diarrhoea) respectively although beneficial require confirming in a large clinical trial. Certainly, a robust double-blinded controlled clinical study is warranted with a more homogeneous group of patients. For example, a study that would include patients that have been diagnosed with the same type of cancer (e.g., large bowel cancer). In this respect to further investigate the overall effect that an adjunctive treatment with a probiotic formulation may have on chemotherapy or radiotherapy and or immunotherapy efficacy. An effect that could reinforce the gut bacterial cohort of commensal bacteria and generated metabolites improving the strength of the intestinal epithelial barrier / scaffold and local mucosal immunity.

## Conclusions and future perspectives

The study confirmed that the gut microbiome was significantly affected by cancer treatments by either radiotherapy, chemotherapy and immunotherapy. Moreover, we posit that the study could provide insight on the role that the intestinal microbiome could play as a marker with critical bacterial components reducing the severity of cancer treatment side effects. Structural intestinal barriers that can be adversely affected the microbiome plays key roles in providing defensive factors that include mucus production and turnover, the control of inflammatory responses and the maintenance of low levels of pathobionts, whilst maintaining tight intestinal epithelial junctions, that overall promote health.

Cancer treatments contribute to intestinal dysbiosis and barrier integrity dysfunction which leads to additional adverse sequalae that can affect treatment progression. Although much work has been done in identifying biomarkers related to the initiation and progression of cancers especially those in the colon, require further investigations. Concomitant effective lifestyle and dietary modifications continue to be critical areas of research that aim to maintain and enhance the intestinal microbiome both for cancer prevention and in clinical management. Dedicated microbiome research will further clarify and establish how the microbiome may positively interact with cancer treatments and how this can be used to improve the prognosis for patients. In this regard further understanding the role of the gut microbiome in reducing the side-effects of cancer treatments, including altered bowel function, treatment-limiting diarrhoea, infections, neutropenia, and febrile mucositis may ensue. Simple interventions such as exercise, increased intake of fibre and resistant starch, food grade prebiotics and multi-strain probiotics need to be investigated further to test their efficacy, safety and clinical relevance in the cancer setting.

Intestinal toxicity can be a cancer-treatment-limiting factor (Sun and Xia 2023), whereas maintaining a healthy gut microbiome may be essential for the efficacy of not only chemotherapeutic agents but also immune therapy with check-point inhibitors. Probiotics may help restore intestinal microbiome balance and improve intestinal barrier homeostasis during cancer treatments (Danis et al. 2022). The cohort of cancer patients in this pilot study were reported to have a high intake of fruits, vegetables, and fibre. Hence, it is possible that this was associated with a healthier microbiome (Wang and Geng 2023) which may have contributed to the tolerance of chemotherapy with long-term survival, enhanced by the co-administration of a multi-strain probiotic.

The future of bacterial consortia and combinations of commensal microbial groups and postbiotics (Rad et al. 2021) have been recently investigated as pharmaceutical grade medicines to enhance the efficacy and response rate of checkpoint inhibitors. What is of scientifically plausible interest is that probiotics have a long association historically, with human hosts and as such ratify their inclusion offering a significant adjunctive therapeutic potential.

## Sponsor

Medlab Clinical Ltd provided the probiotic capsules.

## Data Availability

All relevant data are within the manuscript.
